# Clinical research progress of umbilical cord blood mesenchymal stem cells in Knee articular cartilage repair: A review

**DOI:** 10.1097/MD.0000000000041402

**Published:** 2025-02-07

**Authors:** ZhongKai Liao

**Affiliations:** a The Second Affiliated Hospital of Hainan Medical College, Master of Medicine, Haikou, China.

**Keywords:** cartilaginous injury, knee osteoarthritis, mesenchymal stem cells

## Abstract

Umbilical cord blood mesenchymal stem cells (UCB-MSCs) are a type of adult stem cell with multipotent differentiation potential and immunoregulatory functions, primarily found in neonatal cord blood. Due to their noninvasive collection method, abundance, and ease of preservation, UCB-MSCs represent a promising biological material. This review examines the clinical research on UCB-MSCs in knee articular cartilage repair, highlighting their regenerative potential for treating knee joint cartilage defects. Our aim is to provide insights into current applications and propose directions for future research, focusing on optimizing clinical use and enhancing patient outcomes.

## 1. Introduction

Knee osteoarthritis (KOA) is a whole joint disease involving all joint tissues. KOA is characterized by subchondral bone remodeling, meniscal degeneration, inflammation, and fibrosis of the infrapatellar fat pad and synovial membrane.^[1]^ It is one of the primary causes of disability in the elderly worldwide. With an accelerating aging population, the prevalence of KOA is anticipated to rise further, leading to an increasingly heavy societal and medical burden as well as a loss of workforce.^[2]^ In China, the incidence of KOA increased from 4.6 to 13.2% between 2008 and 2017, with a higher prevalence among women and in urban areas.^[3]^ The pathogenesis of cartilage damage in the knee joint is not entirely understood but may be related to various factors such as mechanical stress, biological elements, free radicals, cytokines, and sex hormones.^[4,5]^ When the joint is overloaded or unevenly distributed, the arch structure of the cartilage matrix is damaged, and the chondrocytes are left unprotected and injured.^[6,7]^ As cartilage degenerates, its mechanical properties, such as stiffness and thickness, are diminished, leading to decreased load-bearing capacity and increased susceptibility to further damage.^[8]^ The deterioration of the extracellular matrix, which is primarily composed of type II collagen and proteoglycans, disrupts the mechanical stability of the cartilage.^[9]^ This results in a higher permeability, which alters the tissue’s ability to absorb and distribute forces effectively, exacerbating the degeneration process.^[9]^ In addition, chondrocytes – the cells responsible for maintaining cartilage homeostasis – experience significant biomechanical stress. The altered mechanical environment in osteoarthritis (OA), characterized by changes in the stiffness and composition of the surrounding cartilage, impacts chondrocyte function and leads to their eventual dysfunction, contributing to disease progression.^[8,10]^ These biomechanical alterations create a feedback loop, where cartilage damage leads to chondrocyte dysfunction, which in turn accelerates cartilage breakdown, resulting in a degenerative cycle commonly seen in OA.^[10]^ Cytokines are produced from inflamed OA tissue, that is, infrapatellar fat pad and synovial membrane.^[11]^ Inflammatory cytokines such as tumor necrosis factor-α and interleukin-1 mediate the increased expression of various proteinases in articular cartilage and synovium, leading to the degradation of the collagen network and proteoglycans in the articular cartilage matrix.^[12]^ In addition, free radicals, estrogen deficiency, and changes in the subchondral bone might also contribute to the progression of cartilage damage.^[13,14]^ The American College of Rheumatology and European League Against Rheumatism guidelines for KOA management emphasize a combination of nonpharmacological and pharmacological interventions to improve patient outcomes.^[15]^ There are numerous treatment methods for KOA, but most are symptomatic treatments, such as various painkillers (including nonsteroidal antiinflammatory drugs), sodium hyaluronate injections, chondroitin sulfate, and glucosamine, which cannot regenerate cartilage.^[16,17]^ In most cases, these merely delay disease progression. Eventually, severe degeneration necessitates surgical interventions like high tibial osteotomy (HTO), unicompartmental knee arthroplasty, or total knee replacement, increasing patients’ socioeconomic burden and discomfort.^[18]^ In recent years, with a deeper understanding of the pathogenesis and treatment methods of KOA, some novel nonsurgical treatment methods have emerged, such as antiinterleukin-1β drugs, platelet-rich plasma, and stem cells.^[19,20]^ These treatments aim to modulate inflammatory responses, stimulate cartilage regeneration, repair cartilage defects, thereby relieving pain, improving function, slowing down the disease or reversing the pathological process.^[21]^ In addition, some new surgical methods, such as knee nerve radiofrequency ablation, robot-assisted knee replacement, and laser therapy, have emerged. These treatments enhance surgical safety and efficacy through precise positioning, excision, and implantation.^[22]^ Nevertheless, the overall management of KOA is still far from satisfactory, becoming one of the significant causes of chronic disability in the elderly.

In recent years, mesenchymal stem cells (MSCs), as a type of stem cell with multi-lineage differentiation potential and immunomodulatory properties, have garnered considerable attention.^[23–25]^ MSCs can be derived from bone marrow, adipose tissue, or umbilical cords, each source having distinct characteristics (Table 1). Following in vitro expansion, these cells can be transplanted into damaged cartilage tissue through intraarticular injection.^[36]^ MSCs can differentiate into chondrocytes and secrete various growth factors and cytokines, promoting the repair and regeneration of cartilage tissue.^[36,37]^ Moreover, research by Yu et al found that mitochondria derived from MSCs could be phagocytosed by cartilage after intraarticular injection, improving pathological cartilage injury, reducing inflammation, inhibiting chondrocyte apoptosis, and promoting mitochondrial biogenesis, thereby improving the KOA phenotype.^[38]^ To date, several clinical trials have confirmed the safety and effectiveness of MSCs in treating KOA,^[39]^ with most adopting autologous bone marrow mesenchymal matrix cells (BM-MSCs). For instance, a randomized Phase I clinical trial by Davatchi et al^[39,40]^ suggested that BM-MSCs could improve patients’ pain, walking, joint mobility, etc. However, these improvements began to decline after 6 months, though they remained better than baseline levels 5 years later. Thus, MSC therapy is a safe and effective method, but better long-term outcomes may require early stage or multiple injections. Chahal et al^[41]^ conducted a randomized, double-blind, placebo-controlled Phase I/II clinical trial assessing the safety and efficacy of BM-MSCs, employing various analysis methods, including patient-reported outcomes, imaging, biomarkers, and molecular fingerprints. They found that autologous BM-MSCs were safe and could significantly improve patients’ pain and symptoms 12 months post-treatment. Their study also revealed that BM-MSCs could reduce synovial inflammation and cartilage degradation, providing a highly predictive donor selection criterion, which is significant for applying MSC therapy in osteoarthritis of the knee. Lamo-Espinosa et al^[42]^ summarized the results analysis of 2 clinical trials using autologous BM-MSCs for treating KOA. They used Huskisson graph to quantify treatment effects and compare different conditions (e.g., cell numbers). They found that using 10,40, or 100 million autologous cells per knee yielded similar healing effects, with the outcomes maintained after 1, 2, and 4 years post a single cell injection. These results show that, in addition to being safe and simple, this cell therapy has significant, durable, and repeatable effects.

**Table 1:** The characteristics of mesenchymal stem cells from different sources.

Type	UCB-MSCs	AD-MSCs	BM-MSCs	References
Source	Umbilical cord blood	Adipose tissue	Bone marrow	^[5,26]^
Source stability	High	Moderate	Moderate	^[5,26]^
Acquisition method	Noninvasive, easy	Invasive, surgical	Invasive, surgical	^[5]^
Cell quantity	Relatively low	Relatively abundant	Relatively abundant	^[5,27]^
Differentiation potential	Moderate	High	High	^[5,26]^
Immunomodulatory effects	Strong	Moderate	Moderate	^[26,27]^
Immunogenicity	Low	Low	Moderate	^[26,27]^
Ethical concerns	None	None	Present	^[5,26]^
Safety	High	High	Low	^[5]^
Cartilage regeneration ability	Limited	Limited	Excellent	^[26]^
Growth factor secretion	Moderate	Moderate	Abundant	^[5,27]^
Cell viability	Relatively low	Relatively high	Relatively high	^[24]^
Clinical potential	Promising	Promising	Widely used	^[4,28–33]^
Purpose	Used for the treatment of heart cerebrovascular disease, liver diseases, bone and muscle degenerative diseases, brain and spinal cord nerve injuries, Alzheimer disease, etc	Used to treat bone defect, cartilage defect, myocardial infarction, diabetes etc	Used in the treatment of hematopoietic system diseases, autoimmune diseases, osteochondral defects	^[25,30–32,34,35]^

AD-MSC = adipose-derived mesenchymal stem cells, BM-MSC = bone marrow mesenchymal matrix cell, UCB-MSC = umbilical cord blood mesenchymal stem cell.

Currently, knee cartilage repair still faces certain limitations and challenges, such as difficulty in achieving complete cartilage regeneration, mismatch of cartilage and matrix, and the ease of degeneration of transplanted cartilage.^[43]^ More effective and safer treatment methods need to be identified. In recent years, MSCs derived from umbilical cord blood, with their multi-lineage differentiation potential, immunomodulatory properties, low immunogenicity, abundant sources, and ease of procurement, have emerged as novel candidate cells for treating knee cartilage damage.^[44]^ Studies have shown that umbilical cord blood MSCs (UCB-MSCs) can differentiate into chondrocytes and integrate with autologous cartilage to form new tissue. Furthermore, UCB-MSCs secrete growth factors that inhibit cartilage matrix degradation and inflammatory responses, thus holding broad potential in knee cartilage repair.^[27,44]^ Therefore, MSCs derived from umbilical cord blood have a broad application prospect in the treatment of knee cartilage damage.

This review is based on a systematic literature search strategy, utilizing databases such as PubMed, Embase, Cochrane Library, Web of Science, and Google Scholar, with the main keywords being “UCB-MSCs,” “KOA,” or “Cartilage repair.” The search focused on English-language studies published between 2000 and 2024, selecting clinical research and reviews related to UCB-MSCs in knee cartilage repair, ensuring that the included literature is scientifically rigorous, comprehensive, and relevant.

## 2. Characteristics of UCB-MSCs

UCB-MSCs are a type of adult stem cell with multi-lineage differentiation potential and immunomodulatory capabilities.^[26,28,34]^ Given suitable induction conditions, they can differentiate into osteocytes, chondrocytes, adipocytes, neurons, myocytes, and other cell types.^[35]^ They primarily exist in umbilical cord blood, umbilical cord, peri-umbilical blood vessels, and Wharton jelly.^[45]^ Among different MSC sources, UCB-MSCs were the earliest MSCs to be isolated from umbilical cord blood and are currently among the most widely applied in clinical settings.^[46]^ UCB-MSCs are sourced from neonatal umbilical cord blood – a noninvasive, abundant, and easily procured and stored biological material.^[45]^ Two primary isolation and cultivation methods exist for UCB-MSCs: direct and indirect methods. MNCs, which include lymphocytes, monocytes, and other immune cells, are isolated from umbilical cord blood as a primary step in the extraction of UCB-MSCs. These cells are crucial because MNCs provide a source of stem cells and progenitor cells that can be further processed to obtain high-purity UCB-MSCs for therapeutic applications.^[29]^ After several fluid exchanges and subcultures, cells characteristic of MSCs are selected. The indirect method first combines the MNCs from umbilical cord blood with fibronectin or other adhesion factors, which are then transferred to the culture dish. By taking advantage of the affinity of MSCs for adhesion factors, MSCs of higher purity can be isolated.^[47]^ The phenotype of UCB-MSCs is similar to that of MSCs from other sources, expressing mesenchymal markers like CD73, CD90,CD105, but not expressing hematopoietic markers like CD34, CD45, and immunogenic molecules such as HLA-DR.^[48]^ In addition, UCB-MSCs possess immunomodulatory properties, inhibiting the activation and proliferation of effector cells such as T cells, B cells, natural killer cells, and dendritic cells, promoting the generation of regulatory T cells, and secreting various antiinflammatory and pro-reparative factors, thereby alleviating tissue damage and inflammatory responses.^[49]^ Compared with MSCs from other sources, UCB-MSCs have several advantages and unique features.^[50]^ First, UCB-MSCs are derived from neonatal umbilical cord blood, free from ethical issues and age restrictions, and abundant in quantity. Second, UCB-MSCs have stronger proliferative capabilities and longer lifespans; during in vitro culture, they can undergo more passages without losing their differentiation potential. Third, UCB-MSCs possess lower immunogenicity and stronger immunomodulatory capabilities, making them less likely to provoke rejection reactions during allogeneic transplantation and suitable as “universal donors.” Fourth, UCB-MSCs exhibit higher plasticity and differentiation potential, capable of differentiating into more diverse cell types and even across germ layers. In summary, UCB-MSCs, with their myriad advantageous characteristics, are a type of stem cell with a broad application prospect in knee cartilage repair.

## 3. Clinical research progress of UCB-MSCs in knee cartilage repair

In recent years, the clinical application of UCB-MSCs in knee cartilage repair has gained attention. Based on our literature search, we identified several representative clinical studies (Table 2).

**Table 2:** Clinical studies of US-MSCs for osteoporosis treatment.

Author	Study design	Outcomes	References
Lee et al.	A retrospective study on patients with unicompartmental medial osteoarthritis and kissing lesions who underwent high tibial osteotomy and microfracture arthroscopy. From January 2015 to December 2019, patients received either BMAC or allogeneic UCB-MSCs to treat full-thickness cartilage defects (≥ICRS grade 3B) in the medial femoral and tibial condyles	The study found that the UCB-MSC treatment was more effective than the BMAC method for cartilage regeneration in patients with medial unicompartmental osteoarthritis of the knee	^[30]^
Ryu et al.	Conducted a comparative, retrospective study on 52 patients with knee cartilage defects, who underwent cartilage repair surgery with either BMAC (25 patients) or UCB-MSCs (27 patients)	Both BMAC and UCB-MSC treatment groups demonstrated significant clinical and MRI-based improvements 2 years post-surgery, with no significant difference observed between the 2 groups	^[31]^
Lim et al.	A randomized controlled clinical trial to compare UCB-MSC-HA implantation to minimally invasive surgery in treating large, full-thickness cartilage defects in elderly patients	The UCB-MSC-HA group showed significantly better cartilage repair grades, histological scores, and clinical scores at 48 weeks, with these benefits persisting over a 5-year follow-up. The study suggests that UCB-MSC-HA implantation is an effective and reliable approach for cartilage regeneration in elderly patients with large cartilage defects	^[32]^
Liu et al.	Three patients with knee osteoarthritis received a single UC-MSC transplantation with a total cell count of 5 to 7 × 10^7^ via intraarticular injection. They were followed up for 3 months post-transplantation to observe clinical outcomes and recovery of knee joint function. The results indicated significant pain relief and improved daily activity ability in the patients	The results indicated significant pain relief and improved daily activity ability in the patients	^[51]^
Wang et al	Evaluated intraarticular injections of human umbilical cord MSCs versus sodium hyaluronate for moderate to severe degenerative knee osteoarthritis	Over 6 months, the MSC group showed significant improvements in joint function and quality of life compared to the control, with sustained benefits up to 6 months post-treatment	^[52]^
Pak et al.	hUCB-MSCs were applied for articular cartilage regeneration in knee osteoarthritis patients with severe, full-thickness defects (>2.0 cm²). Among 253 patients, 64 underwent a second-look arthroscopy after 1 year	showing significant improvements in IKDC, WOMAC, and VAS scores. The cartilage regeneration was graded favorably with the Oswestry Arthroscopy Score, with substantial functional and pain relief benefits, suggesting hUCB-MSCs as a promising treatment for younger patients needing alternatives to knee replacement	^[53]^

BMAC = bone marrow aspirate concentrate, hUCB-MSC = human umbilical cord blood-derived mesenchymal stem cell, ICRS = International Cartilage Repair Society, IKDC = International Knee Documentation Committee, MRI = magnetic resonance imaging, MSC = mesenchymal stem cell, UCB-MSC = umbilical cord blood mesenchymal stem cell, UCB-MSC-HA = umbilical cord blood mesenchymal stem cells with hyaluronic acid, VAS = Visual Analog Scale, WOMAC = Western Ontario and McMaster Universities Arthritis Index.

Lee et al^[30]^ investigated cartilage regeneration using UCB-MSCs or bone marrow aspirate concentrate (BMAC) in HTO. They retrospectively studied patients with unicompartmental medial OA and kissing lesions who received HTO and microfracture arthroscopy combined with BMAC or allogeneic UCB-MSCs treatment from January 2015 to December 2019, revealing full-thickness cartilage defects (≥International Cartilage Repair Society grade 3B) of the medial femoral and tibial condyles. The study compared the effects of the 2 methods on cartilage repair in patients with medial unicompartmental OA of the knee. Results demonstrated that, regardless of the treatment method used, the UCB-MSC method was more effective than the BMAC method in cartilage regenerative treatment for medial unicompartmental osteoarthritis of the knee, although both methods improved the clinical condition of the patients.

Ryu et al^[31]^ conducted a comparative study of BMAC and UCB-MSC implantation in the treatment of knee cartilage defects, evaluating the clinical and magnetic resonance imaging (MRI) outcomes of knee osteoarthritis treatment with 2 different types of MSC implantation. They retrospectively evaluated 52 patients who underwent cartilage repair surgery with BMAC (25 patients) and UCB-MSC (27 patients) 2 years post-surgery. Clinical outcomes were assessed using Visual Analog Scale, International Knee Documentation Committee subjective score, and Knee Injury and Osteoarthritis Outcome Score. Cartilage repair was assessed using the Magnetic resonance observation of cartilage repair tissue score and modified MRI assessment based on the International Cartilage Repair Society cartilage repair rating system. Both groups demonstrated significant clinical and MRI improvements at the 2-year follow-up, but no significant differences were found between the 2 groups. The study suggested that BMAC or UCB-MSC implantation is a safe and effective method for treating knee cartilage defects, but more cases and long-term prospective studies are still needed.

Lim et al^[32]^ conducted a randomized controlled clinical trial comparing the effects of UCB-MSC-HA implantation and minimally invasive surgery for the treatment of large, full-thickness cartilage defects in elderly patients. Results showed that the UCB-MSC-HA implantation group had superior cartilage repair grades, histological scores, and clinical scores at 48 weeks compared with the minimally invasive surgery group, and these improvements were maintained over a 5-year follow-up. This study indicates that UCB-MSC-HA implantation is a reliable method for cartilage regeneration, suitable for treating large, full-thickness cartilage defects in elderly patients.

Liu et al^[51]^ conducted a clinical research of 3 patients with knee osteoarthritis received a single UC-MSC transplantation with a total cell count of 5 to 7 × 10^7^ via intraarticular injection. They were followed up for 3 months post-transplantation to observe clinical outcomes and recovery of knee joint function. The results indicated significant pain relief and improved daily activity ability in the patients. No significant differences were observed in biochemical parameters, including blood routine, liver function, and kidney function, before and after treatment. The study concluded that intraarticular injection of UC-MSC transplantation could ameliorate clinical symptoms and delay the progression of osteoarthritis, potentially offering a safe and effective treatment option. However, further observation is necessary to evaluate its long-term efficacy.

In a randomized controlled trial, Wang et al^[52]^ evaluated intraarticular injections of human umbilical cord MSCs versus sodium hyaluronate for moderate to severe degenerative knee osteoarthritis. Over 6 months, the MSC group showed significant improvements in joint function and quality of life compared with the control, with sustained benefits up to 6 months post-treatment. Although pain and swelling were more common in the MSC group initially, their Lysholm, Western Ontario and McMaster Universities Arthritis Index, and SF-36 scores showed lasting improvements, indicating MSC injections as an effective option for osteoarthritis symptom relief.

In a study by Pak,^[53]^ human umbilical cord blood-derived MSCs were applied for articular cartilage regeneration in knee osteoarthritis patients with severe, full-thickness defects (>2.0 cm²). Among 253 patients, 64 underwent a second-look arthroscopy after 1 year, showing significant improvements in International Knee Documentation Committee, Western Ontario and McMaster Universities Arthritis Index, and Visual Analog Scale scores. The cartilage regeneration was graded favorably with the Oswestry Arthroscopy Score, with substantial functional and pain relief benefits, suggesting human umbilical cord blood-derived MSCs as a promising treatment for younger patients needing alternatives to knee replacement.

## 4. Analysis of the clinical efficacy and safety of UCB-MSCs

Based on the aforementioned clinical studies, we can analyze the clinical efficacy and safety of UCB-MSCs in knee cartilage repair as follows.

UCB-MSC transplantation promotes knee cartilage regeneration, improves joint function, reduces pain, and enhances quality of life.^[54]^ Compared with autologous chondrocyte transplantation or microfracture surgery, UCB-MSC transplantation has similar or superior effects, especially demonstrating significant benefits in elderly patients.^[55]^ This might be associated with the high proliferative capacity, differentiation potential, stability, and plasticity of UCB-MSCs.^[56]^ In addition, UCB-MSC transplantation can enhance cartilage repair through various mechanisms, such as regulating immune responses, inhibiting inflammation, promoting angiogenesis, and stimulating chondrocyte proliferation and differentiation, by secreting a variety of growth factors, cytokines, and exosomes.^[57]^

In terms of safety, no serious adverse events such as infections, tumors, or immune rejection have occurred in the current clinical research on UCB-MSC transplantation, indicating good safety and tolerability. This might be related to the characteristics of UCB-MSCs, such as low immunogenicity, high immune-regulatory capacity, and absence of ethical controversy.^[56]^ However, current clinical studies lack adequate safety monitoring and reporting, so further reinforcement of safety assessment and monitoring for UCB-MSC transplantation is needed.

## 5. Issues and limitations in the clinical application of UCB-MSCs

Despite the promising prospects of UCB-MSCs in knee cartilage repair, several limitations remain.

First, the definition and identification standards of UCB-MSCs are not uniform, and different culture conditions and treatment methods may affect the quantity, quality, differentiation ability, and functional characteristics of UCB-MSCs. Therefore, it is necessary to establish more scientific, accurate, reliable, and reproducible definition and identification standards for UCB-MSCs.^[58]^ Currently, there is no international consensus on the definition and identification standards for UCB-MSCs. Methods used by different institutions vary, leading to challenges in quality control and comparability.^[33]^ Moreover, UCB-MSCs may undergo phenotypic changes, functional decline, and genetic variations during in vitro culture, affecting their stability and predictability.^[59]^ Therefore, a more stringent and standardized UCB-MSC quality control system needs to be established, including various aspects such as cell origin, isolation purification, expansion culture, cryopreservation and transportation, phenotypic identification, and functional evaluation, to ensure the safety and effectiveness of UCB-MSCs.

Second, the optimal dosage, route, and timing of UCB-MSCs are still unclear. Different choices may affect the homing, survival, differentiation, and function of UCB-MSCs in vivo, further research is needed to determine the optimal dosage, route, and timing for UCB-MSC transplantation.^[60]^ Currently, there is no unified standard for the optimal transplantation scheme of UCB-MSCs in knee joint cartilage repair. Different studies have used different dosages (from 10^6^ to 10^8^ cells), routes (from intraarticular injection to surgical implantation), and timings (from acute injury to chronic degeneration), leading to differences in transplant effects. In addition, the homing, survival, differentiation, and function of UCB-MSCs in vivo are influenced by various factors, such as recipient tissue microenvironment, immune response, and hemodynamics. Therefore, personalized UCB-MSC transplantation schemes should be developed based on different injury types, degrees, locations, and other factors, along with long-term follow-ups and evaluations.

Third, the mechanism of action of UCB-MSCs remains unclear. Further studies are needed to understand their interactions with damaged cartilage cells, surrounding cells, and molecules, as well as their regulation of cartilage metabolism, inflammation, repair, and regeneration.^[61]^ Currently, there are many uncertainties and disputes about the mechanism of UCB-MSCs in knee joint cartilage repair. On 1 hand, there is no definitive evidence that UCB-MSCs can directly differentiate into cartilage cells and participate in cartilage regeneration. On the other hand, whether UCB-MSCs can release a variety of bioactive molecules, such as growth factors, cytokines, exosomes, etc, through paracrine secretion, affecting the proliferation, differentiation, migration, apoptosis, and other behaviors of damaged cartilage cells and surrounding cells, and regulating cartilage metabolism, inflammation, repair, and regeneration processes needs more experimental validation. Moreover, the in vivo mechanism of UCB-MSCs might be regulated by many factors, such as the origin, phenotype, dose, route, timing of UCB-MSCs, and the state, changes, and feedback of the recipient tissue microenvironment. Therefore, more precise and comprehensive models of UCB-MSC mechanisms of action need to be established and validated from multiple levels and perspectives.

Fourth, the long-term effects and safety of UCB-MSCs are still uncertain. Whether UCB-MSCs will undergo aging, mutation, transformation, migration, and other phenomena in vivo, whether they will cause immune reactions, tumor formation, infection transmission, and other complications, whether they will continuously improve the structure and function of joint cartilage, and whether they will delay or reverse degenerative changes in the joints all need to be followed up and observed for a longer time.^[62]^ Currently, there is a lack of sufficient data to support the long-term effects and safety of UCB-MSCs in knee joint cartilage repair. Most clinical studies have only conducted short-term follow-ups and evaluations (6 months to 2 years), finding that UCB-MSC transplantation can improve patients’ symptoms and function and did not find any serious adverse events.^[30–32]^ However, long-term effectiveness and safety of UCB-MSC transplantation (>5 years) remain uncertain. Potential concerns include in vivo aging or mutation of UCB-MSCs, malignant transformation, immune reactions, pathogen transmission, and the ability to maintain cartilage structure and function over time. Therefore, longer follow-ups and observations are needed, and more sensitive and precise detection methods should be used to evaluate the long-term effects and safety of UCB-MSC transplantation in knee joint cartilage repair.

## 6. Conclusion and outlook

UCB-MSCs are a promising stem cell source with multiple biological functions, easily accessible, and free from ethical controversies.^[28]^ They possess certain advantages such as easy accessibility, good immune compatibility, and lack of ethical controversy.^[63]^ The clinical application of UCB-MSCs in the repair of knee joint cartilage represents an emerging therapeutic strategy, possessing considerable strengths and potential, but also faces some challenges and questions. This article has summarized the research progress of UCB-MSCs in the repair of knee joint cartilage, mainly including the following aspects.

First, the clinical effect of UCB-MSCs in the repair of knee joint cartilage is definite. Numerous clinical studies have shown that UCB-MSCs transplantation promotes cartilage regeneration in the damaged areas of knee joints, improves patients’ joint function and quality of life, and alleviates pain and discomfort, showing promising outcomes and safety. This is related to the fact that UCB-MSCs have the potential to differentiate into cartilage cells, exhibit antiinflammatory and immune-regulatory mechanisms. UCB-MSCs can migrate to the damaged cartilage tissue and differentiate into cartilage cells to repair the deficient cartilage. UCB-MSCs can also secrete a variety of bioactive molecules, such as growth factors, cytokines, exosomes, etc, affecting the proliferation, differentiation, migration, apoptosis of damaged cartilage cells and surrounding cells, and regulating the processes of cartilage metabolism, inflammation, repair, and regeneration. Furthermore, UCB-MSCs can inhibit the body’s immune rejection or allergic reactions to allogeneic cells and promote the body’s tolerance to its own tissues.

Second, there are still some problems and deficiencies in the application of UCB-MSCs in the repair of knee joint cartilage. Current clinical research has some limitations, such as small sample size, short follow-up time, lack of unified evaluation standards, and objective indicators. Hence, more high-quality, large-sample, long-term follow-up, multi-center, randomized controlled clinical trials are needed to verify their clinical efficacy and safety. Furthermore, it is necessary to further explore the definition and identification standards, optimal transplantation scheme, mechanism of action, long-term effects, and safety issues of UCB-MSCs to provide more scientific, accurate, reliable, and reproducible evidence for the clinical application of UCB-MSCs in the repair of knee joint cartilage. Currently, there is no international consensus on the definition and identification standards for UCB-MSCs. Methods used by different institutions vary, leading to challenges in quality control and comparability. UCB-MSCs may undergo phenotypic changes, functional decline, and genetic variations during in vitro culture, affecting their stability and predictability. The optimal dosage, route, and timing of UCB-MSCs are still unclear. Different choices may affect the homing, survival, differentiation, and function of UCB-MSCs in vivo. The mechanism of action of UCB-MSCs is still not clear, and further research is required to understand how UCB-MSCs interact with damaged cartilage cells, surrounding cells, and molecules in the body, how they regulate the metabolism, inflammation, repair, and regeneration processes of cartilage, and how they affect joint stability, function, and pathology. The long-term effects and safety of UCB-MSCs are still uncertain. More extended follow-ups and observations are needed to determine whether UCB-MSCs undergo aging, mutation, transformation, migration in vivo, whether they cause immune reactions, tumor formation, infection transmission, whether they continuously improve the structure and function of joint cartilage, and whether they delay or reverse degenerative changes in the joints.

Last, although UCB-MSCs face challenges such as technical complexity and long-term safety concerns in knee cartilage repair, they have broad potential in regenerative medicine, particularly with innovations in gene editing and tissue engineering. With the development of stem cell technology and tissue engineering technology, UCB-MSCs may make more innovations and breakthroughs in the repair of knee joint cartilage. For instance, technologies such as stem cell exosomes, microencapsulation of stem cells, and gene editing of stem cells can enhance the bioactivity and functional characteristics of UCB-MSCs, enhancing their role in cartilage repair.^[64]^ Materials such as acellular cartilage extracellular matrix, nanofibers, and hydrogels can be used to construct a more suitable carrier and scaffold for UCB-MSCs, improving their homing, survival, and differentiation in cartilage repair.^[64]^ Multimodal imaging, biomarkers, histology, etc, can be used to more objectively and accurately evaluate the effects and safety of UCB-MSCs in cartilage repair.^[65]^ At the same time, it is necessary to strengthen the research and standardization of UCB-MSCs in the repair of knee joint cartilage to ensure their rationality and legality in clinical applications.

In summary, UCB-MSC transplantation as a novel method for repairing knee joint cartilage has shown promising results and safety in clinical settings, providing an effective treatment option for patients with knee joint cartilage injuries. However, this method is still in its early stages of exploration and requires more high-quality, large-sample, randomized controlled, multi-center clinical trials to validate its long-term effects and safety and explore indications, optimal dosages, optimal time windows, and other related issues. We look forward to more innovative breakthroughs in the application of UCB-MSCs in the repair of knee joint cartilage, which will bring better treatment outcomes and quality of life for patients.

## Author contributions

**Writing – original draft:** Zhongkai Liao.

**Writing – review & editing:** Zhongkai Liao.
